# Advances in siRNA therapeutics and synergistic effect on siRNA activity using emerging dual ribose modifications

**DOI:** 10.1080/15476286.2022.2052641

**Published:** 2022-03-30

**Authors:** Sumit Gangopadhyay, Kiran R Gore

**Affiliations:** Department of Chemistry, Indian Institute of Technology Kharagpur, Kharagpur, India

**Keywords:** siRNA, sugar modification, vinylphosphonate, gene silencing, RNAi clinical trial

## Abstract

Nucleic acid-based therapeutics that control gene expression have been steadily progressing towards achieving their full clinical potential throughout the last few decades. Rapid progress has been achieved in RNAi-based therapy by optimizing high specificity and gene silencing efficiency using chemically modified siRNAs. Since 2018, four siRNA drugs – patisiran, givosiran, lumasiran, and inclisiran, were approved by the US FDA, providing a testament to the promise of RNAi therapeutics. Despite these promising results, safe and efficient siRNA delivery at the target site remains a major obstacle for efficient siRNA-based therapeutics. In this review, we have outlined the synergistic effects of emerging dual ribose modifications, including 2’,4’- and 2’,5’-modifications, 5’-*E/Z-*vinylphosphonate, and northern methanocarbacyclic (NMC) modifications that have contributed to drug-like effects in siRNA. These modifications enhance nuclease stability, prolong gene silencing efficiency, improve thermal stability, and exhibit high tissue accumulation. We also highlight the current progress in siRNA clinical trials. This review will help to understand the potential effects of dual ribose modifications and provides alternative ways to use extensive 2’-modifications in siRNA drugs. Moreover, the minimal number of these dual ribose modifications could be sufficient to achieve the desired therapeutic effect. In future, detailed *in vivo* studies using these dual ribose modifications could help to improve the therapeutic effects of siRNA. Rational design could further open doors for the rapid progress in siRNA therapeutics.

**Figure:**
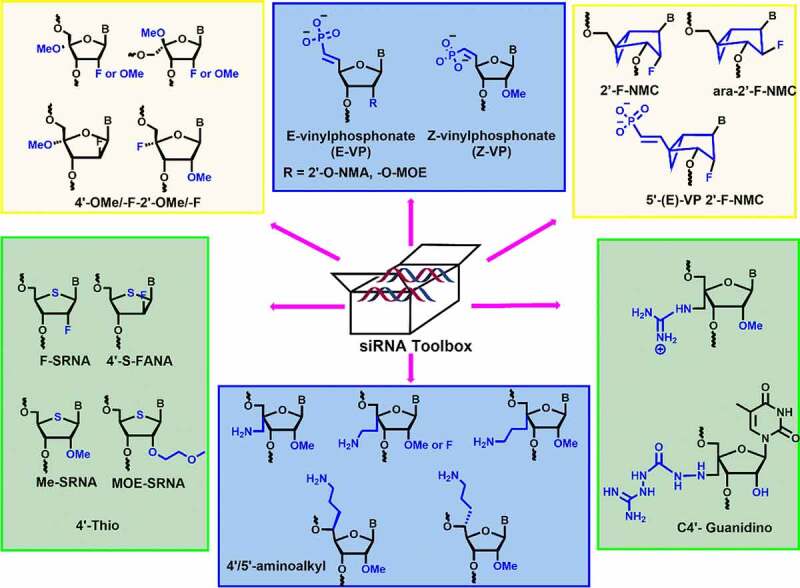

## 1. Introduction

The discovery of RNA interference (RNAi) in 1998 by Fire and Mello is a highly promising tool for gene therapy [1,2]. Nucleic acid therapeutics including small interfering RNA (siRNA), microRNA (miRNA), and antisense oligonucleotides (AONs) are potential gene silencing platforms, which have shown the promising results against a broad spectrum of diseases [3,4].

The endogenous RNAi mechanism begins with the cleavage of double-stranded RNA (dsRNA) into 21–22 base pairs nucleotide sequence by the enzyme, dicer [5]. This siRNA consists of sense (passenger) and antisense (guide) strands [6,7]. The siRNAs combine with argonaute-2 (Ago-2) and other supplementary protein residues to form the RNA-induced silencing complex (RISC) [8]. The activated RISC is comprised of the guide strand that binds specifically to the target sequence of mRNA. Ago2 cleaves the mRNA to achieve an efficient gene silencing [9]. RNAi-based therapeutics are the most efficient and reliable tool to achieve gene silencing of the desired target gene. Many diseases including cancers, neurodegenerative diseases, autoimmune diseases, and viral infection have been explored using siRNA-based therapeutics [10,11].

Despite the aforementioned remarkable success, there are several challenges associated with the siRNA therapeutics, including their limited stability against nucleases, off-target effects, targeted delivery to a particular tissue or organ and unfavourable drug-like properties 12–17 [12-17]. Along the routes to address these challenges, chemical modifications significantly contributed to drug-like properties of siRNAs and played an invaluable role in their clinical success. Interestingly, all recently approved siRNA drugs were extensively modified with ribose 2’-modifications. In the last two decades, several emerging dual ribose modifications have shown strong influence on the physicochemical properties and overall efficacy of siRNA.

The synergistic effects of dual modifications including 2’,4’-, 2’,5’-modifications, 5’-*E/Z-*vinylphosphonate, and northern methanocarbacyclic (NMC) modifications showed enhanced nuclease stability and prolonged silencing activity in comparison to mono modifications 11–13[11-13]. We have categorized this review into two major sections. In the first section, we summarize the current status on siRNA clinical trials and the significance of 2’-modifications in these drugs. In the second part, we discuss the emerging dual ribose modifications employed in siRNAs and highlight the key results. From these results, it is clear that the minimal number of dual ribose modifications could be sufficient to achieve the maximum therapeutic effect and could be a potential alternative to mono modifications.

## 2. Overview of clinical trials

The siRNA drugs took almost 20 years to reach the market since the discovery of RNAi. Since 2018, four siRNA drugs from Alnylam pharmaceuticals – patisiran, givosiran, lumasiran, and inclisiranhave been approved by the US. FDA [11]. Moreover, seven siRNA drugs are currently in advanced stages of clinical trials. Patisiran (ONPATTRO) is the first siRNA drug approved in 2018 for the treatment of polyneuropathy of hereditary transthyretin-mediated (hATTR) amyloidosis [18]. Patisiran contains eleven 2’-methoxy (2’-*O*-Me) modifications (two modifications in the guide strand and nine in the passenger stand) and 2′-deoxy thymidine modifications at the 3’-end of both the strands.

**Figure 1.**
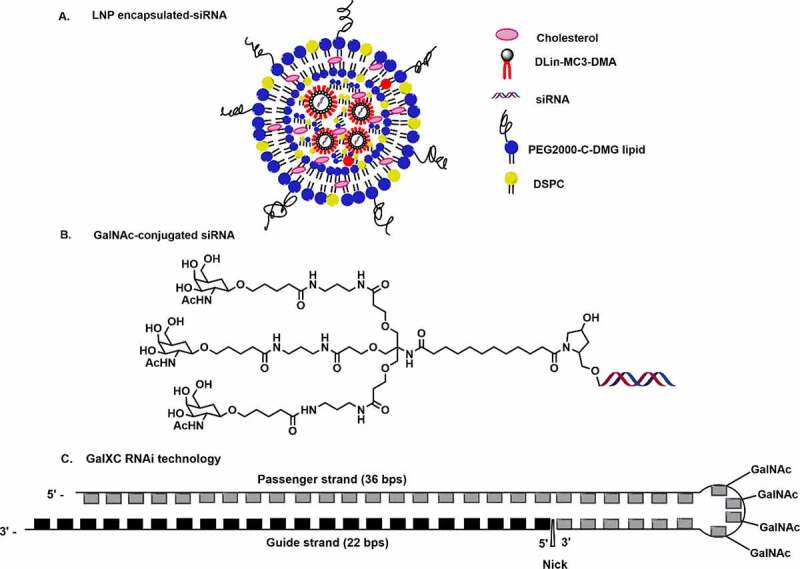
Delivery platforms in siRNAs drugs in advance clinical trials. A. lipid nanoparticle (LNP) encapsulated siRNA B. GalNAc conjugation C. GalXC RNAi technology.

Patisiran is based upon the second-generation lipid nanoparticle (LNP) formulation [Figure 1] which includes cholesterol, 1,2-distearoyl-sn-glycero-3-phosphocholine (DSPC), (*R*)-2,3-bis(octadecyloxy)propyl-1-(methoxy polyethylene glycol 2000) carbamate (PEG2000-C-DMG), and an ionizable amino lipid (6*Z*,9*Z*,28*Z*,31*Z*)-heptatriaconta-6,9,28,31-tetraen-19-yl-4-(dimethylamino) butanoate (DLin-MC3-DMA) [19]. The LNP formulated patisiran achieves liver-specific delivery of siRNAs via apolipoprotein E (ApoE) receptor endocytosis [20,21]. It binds to the 3ʹ-untranslated region (3’-UTR) of both wild type (WT) as well as mutated TTR mRNA and cleaves it which prevents the TTR protein expression. In June-2021, APOLLO-B was enrolled for phase III study of patisiran for the treatment of TTR-mediated Amyloidosis (ATTR) with patients having cardiomyopathy [22]. Similar to patisiran, vutrisiran is another siRNA candidate targeting TTR mRNA and is currently being evaluated in the late stages of phase 3 clinical trial [Table 1].

**Table 1.** siRNA-based drugs in advanced clinical trials

S. no.	SiRNA drugs	Chemical modification	Delivery system/ targeting ligand	Targeting gene	Disease	Company	Phases
1	ONPATTRO (Patisiran)	2′-*O*-Me, dT	LNP-siRNA	TTR	TTR-mediated amyloidosis	Alnylam	Approved (2018)
2	GIVLAARI (Givosiran)	PS, 2′-*O*-Me, 2′-F	GalNAc-siRNA	ALAS1	Acute hepatic porphyria	Alnylam	Approved (2019)
3	OXLUMO (Lumasiran)	PS, 2′-*O*-Me, 2′-F	GalNAc-siRNA	HAO1	Primary hyperoxaluria type 1	Alnylam	Approved (2020)
4	LEQVIO (Inclisiran)	PS, 2′-F, 2′-*O*-Me, dT	GalNAc-siRNA	PCSK9	Hypercholesterolemia	Alnylam Novartis	Approved (2021)
5	Vutrisiran (ALN-TTRSC02)	PS, 2′-*O*-Me, 2′-F	GalNAc-siRNA	TTR	TTR-mediated amyloidosis	Alnylam	Phase III
6	Fitusiran (ALN-AT3SC)	PS, 2′-*O*-Me, 2′-F	GalNAc-siRNA	AT	Haemophilia A and B and rare blood disorders	Alnylam Sanofi Genzyme	Phase III
7	Nedosiran (DCR-PHXC)	PS, 2′-*O*-Me, 2′-F	GalNAc-siRNA	LDHA	Primary hyperoxaluria	Alnylam Dicerna	Phase III
8	Teprasiran (QPI-1002)	2′-*O*-Me	None	p53	Acute kidney injury	Quark Novartis	Phase III
9	Cosdosiran (QPI-1007)	2′-*O*-Me	None	Caspase 2	NAION and glaucoma	Quark	Phase III
10	Tivanisiran (SYL1001)	None	None	TRPV1	Ocular pain and dry eye disease	Sylentis	Phase III

*abbreviations* 2′-F, 2′-fluoro; 2′-*O*-Me, 2′-methoxy; 2′-*O*-MOE, 2’-methoxyethyl; dT, 2’-deoxythymidine; TTR, transthyretin; PS, phosphorothioate linkage; GalNAc, N-acetyl-D-galactosamine; TTR, Transthyretin; LNP, lipid nanoparticle; ALAS1, delta-aminolevulinate synthase 1; LDHA, lactate dehydrogenase A; HAO1, hydroxy acid oxidase 1; EU, European Union; PCSK9, proprotein convertase subtilisin/Kexin type 9; AT, antithrombin; TRPV1, transient receptor potential vanilloid 1; NAION, non-arteritic ischemic optic neuropathy; p53, tumor protein p53

The development of GalNAc conjugation for targeted delivery of siRNA to the liver has greatly advanced siRNA therapeutics. The GalNAc delivery method shows high specificity towards the asialoglycoprotein receptor (ASGPR) present on the surface of the hepatocytes [Figure 1]. In comparison to LNP, the GalNAc platform is preferred due to its straightforward synthetic route, high cell uptake, rapid absorption, high abundance of ASGPR of hepatocytes, and encouraging toxicity profile [23,24]. Vutrisiran is loaded with 2’-fluoro (2’-F), 2’-methoxy (2’-*O*-Me), and phosphorothioate (PS) modifications, and utilizes the GalNAc delivery platform [25]. Recently, the US FDA has accepted the New Drug Application (NDA) for vutrisiran based on the positive results of its phase III study (HELIOS-A) against polyneuropathy of hATTR amyloidosis [26]. In August 2021, vutrisiran was enrolled in the phase III study (HELIOS-B) for the treatment of TTR-mediated Amyloidosis with patients having cardiomyopathy [27]. Unlike Patisiran, GalNAc conjugated siRNA drugs can be effectively delivered by subcutaneous injection rather than the tedious intravenous administration [25]. Provided FDA approval, vutrisiran could be a potential alternative to patisiran in the treatment of hATTR disease.

Givosiran (GIVLAARI or ALN-AS1) is another GalNAc conjugated siRNA drug that was approved by the FDA in November-2019 for the treatment of acute hepatic porphyria (AHP) [28]. Targeting the aminolevulinic acid synthase 1 (ALAS1) gene using givosiran could achieve a significant reduction in ALAS1 expression, thereby normalizing the level of neurotoxic metabolites, including delta-aminolevulinic acid and porphobilinogen [29,30]. GalNAc conjugated givosiran binds strongly to the asialoglycoprotein receptor (ASGPR) present on the surface of the hepatocytes to achieve targeted delivery [24,31].

**Figure 2.**
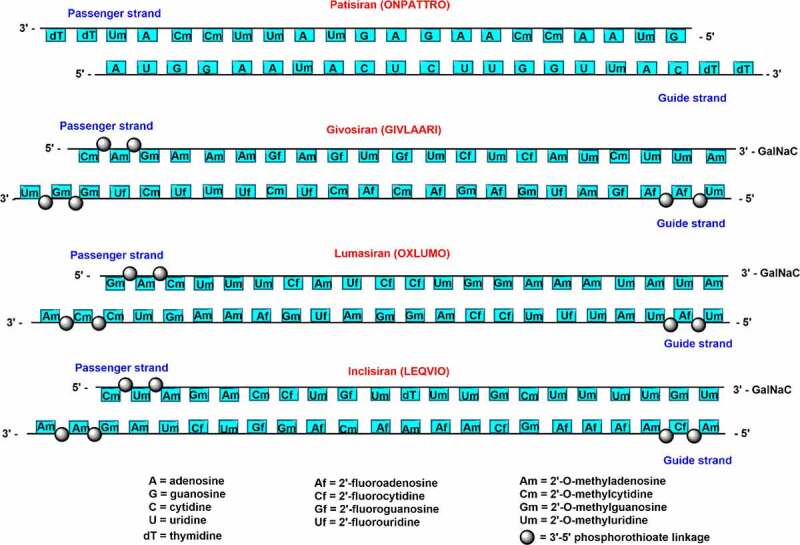
Schematic representation of chemically modified siRNA strands for clinically approved siRNA drugs.

Givosiran consists of 2’-fluoro, 2’-*O*-Me, and six PS modifications at ends of both the strands **[**Figure 2**]**. The GalNac conjugation at 3’-end of passenger strands allows liver-specific delivery, which is the major site for ALAS1 production [28,29]. A schematic representation of the passenger and guide strands with proper chemical modifications of all the approved chemically modified siRNA drugs are summarized in Figure 2. Lumasiran (OXLUMO) or ALN-GO1 is the third siRNA drug approved by the U.S. FDA for the treatment of primary hyperoxaluria type 1 (PH1)-a rare genetic disorder that targets hydroxy acid oxidase 1 (HAO1) [32,33]. PH1 is normally caused by the excessive production of oxalates in the liver. Lumasiran also utilized the GalNAc platform to achieve liver-specific delivery. Lumasiran is comprised of 2’-fluoro, 2’-*O*-Me, and PS modifications at the ends of the strands [10]. The U.S. FDA has approved lumasiran against PH1 which helps to minimize the urinary oxalate level in paediatric and adult patients [34]. The European Medicines Agency (EMA) has approved the use of lumasiran for the treatment of PH1 in all age groups [34]. Recently, the enrolment process for the ILLUMINATE-C Phase 3 study of lumasiran was completed for the treatment of adult and paediatric patients with advanced PH1. ILLUMINATE clinical trials include PH1 patients of all age groups, with advanced renal diseases, including patients on haemodialysis [34].

Like lumasiran, nedosiran (DCR-PHXC) developed by Dicerna pharmaceuticals, targets lactate dehydrogenase A (LDHA) mRNA in the treatment of primary hyperoxaluria (PH) types 1, 2, and 3 [30,35]. Similar to lumasiran<apos;>s GalNAc platform, Dicerna developed the proprietary GalXC delivery platform for nedosiran. Nedosiran is currently being evaluated in the late stages of the phase 3 trial and is the potential therapeutic agent for all the three genetically defined PH subtypes [30,36]. The PHYOX2 clinical trials of nedosiran, including patients with PH1 and PH2, found that nedosiran could be a potential therapeutic option for patients with primary hyperoxaluria 1 [37]. Recently, Dicerna initiated the PHYOX4 clinical study of nedosiran which is a broader version of the PHYOX clinical trial programme for the treatment of PH3 patients with chronic kidney disease [38].

Inclisiran (LEQVIO) or ALN-PCSSC is the first siRNA drug against hypercholesterolemia approved by the FDA in December 2021 [11]. It is a completely modified siRNA drug comprised 2’-deoxy, 2′-fluoro, and 2′-*O*-methyl substituents with six terminal PS linkage modifications **[**Figure 2**]** [39,40]. GalNAc conjugated inclisiran targets the proprotein convertase subtilisin/Kexin type 9 (PCSK9) mRNA that helps to prevent the PCSK9 protein production and upregulate the low-density lipoprotein (LDL)-cholesterol receptor level. Inclisiran siRNA drug could decrease the cardiovascular disease risk by reducing the LDL-cholesterol level [41]. Givosiran, vutrisiran, lumasiran, nedosiran, etc. have been administered subcutaneously by employing the GalNAc conjugation platform which has improved its absorption by hepatocytes, half-life, and overall efficacy [30].

Another GalNAc-conjugated siRNA drug, fitusiran was evaluated in phase III trials. It downregulates antithrombin III (AT) protein production by targeting the SERPINC1 gene in the treatment of patients suffering from haemophilia and rare bleeding disorders (RBDs) [42]. The subcutaneous administration of fitusiran inhibits the production of AT, a protein that inhibits blood clotting. Inhibition of AT subsequently upregulates thrombin generation and decreases the chances of bleeding events [42,43]. Fitusiran is a completely modified siRNA drug comprised 2′-fluoro and 2′-*O*-methyl as well as six terminal PS linkage modifications [30]. In January 2021, the U. S. FDA and the Japanese Pharmaceuticals and Medical Devices Agency (PMDA) have approved the revised dosing plan of fitusiran [44].

Teprasiran (QPI-1002 or I5NP), under phase III clinical trials by Quark Pharmaceuticals along with Novartis, suppresses the expression of the pro-apoptotic gene p53 [30,45]. QPI-1002 is a partially modified siRNA drug under phase III clinical trial for the prevention of major adverse kidney events and the treatment of acute kidney injury (AKI) associated with cardiac surgery. QPI-1002 is a naked, blunt, and unformulated siRNA duplex that consists of 19 nucleotides in both the strands with alternate 2’-*O*-Me modifications. QPI-1002 is the first systematically administered siRNA drug being tested in clinical trials. Teprasiran has a very low plasma half-life resulting in its rapid clearance and facilitates renal filtration. It gets reabsorbed into the kidney through proximal tubular cells [45]. In September 2021, a study was published based on a phase III randomized clinical trial study in which it showed that the severity and duration of early AKI to be reduced significantly in a high-risk patient undergoing cardiac surgery upon teprasiran administration. Teprasiran inhibits the expression of the p53 gene and allows to repair the injured renal tubule cells [46].

Cosdosiran (QPI-1007), developed by Quark Pharmaceuticals for nonarteritic anterior ischemic optic neuropathy (NAION) and primary angle glaucoma [45,47]. QPI-1007 inhibits the expression of caspase 2 by targeting caspase 2 mRNA and protects the loss of retinal ganglion cells in optic neuropathies. QPI-1007, comprised of 19 nucleotides guide and passenger strands, is a partially modified siRNA and consists of 2’-*O*-Me substituents at various positions in the guide strand. An inverted deoxyabasic moiety and an L-DNA cytidine nucleotide are present at the 5’-end of the passenger strand [47]. Interestingly, the intravitreal administration of QPI-1007 into the retina/choroid in rabbits showed much slower renal filtration compared to intravenous administration in the rat, resulting in its rapid clearance [47].

Tivanisiran (SYL1001) is a completely unmodified siRNA drug candidate tested for the treatment of ocular pain and dry eye disease [30,45]. This naked siRNA, SYL1001 targets transient receptor potential cation channel subfamily V member 1 (TRPV1) mRNA, which leads to the knockdown of the capsaicin receptor, present on the ocular surface. Recently, the US FDA authorized phase III trial in which a combination of SYL1001_V with tivanisiran ophthalmic solution were tested against dry eye conditions associated with Sjögren<apos;>s Syndrome [48].

Many of the siRNA drug candidates in various phases of clinical trials have been designed with chemical modification platforms, such as standard template chemistry, enhanced stabilization chemistry (ESC), advanced ESC, and ESC+ [10,25,30]. These chemical modifications have been known to improve the therapeutic aspects, such as metabolic stability, biodistribution, pharmacokinetics, and pharmacodynamic properties of siRNA. Moreover, apart from therapeutic applications, chemically modified oligonucleotides are used for various biochemical and biophysical applications [49].

## 3. Overview of clinically appealing C2’ and non-canonical sugar modifications

Sugar moieties in DNA and RNA differ at the C-2’ position in the furanose sugar. Ribofuranose is a non-planar and puckered ring structure where O3’ and O5’ are linked to the phosphodiester bond in the nucleic acid structure [50]. Sugar 2’-modifications have been extensively studied and found to be most suitable in improving the efficacy of siRNAs. Various ribose 2*’*-modifications, including 2’-*O*-methyl (2’-*O*-Me), 2’-fluoro (2’-F), 2’-methoxyethyl (2’-*O*-MOE) prefer C3’-*endo* conformation and provides high thermodynamic stability in RNA duplexes 51–53 [51-53].

2’-*O*-Me and 2’-F in combination with PS modification have been employed in recently approved siRNA drugs, like givosiran, lumasiran, and inclisiran. The 2’-*O*-Me modification was found to improve serum stability, binding affinity, as well as reduce the innate immune response [51]. However, fully modified siRNA strands with 2’-*O*-Me modifications are non-functional [51]. Similarly, fully modified 2’-F siRNAs have lower RNAi activity than unmodified siRNAs [51]. Another shortfall related to 2’-F modified siRNAs reported as its limited nuclease resistance as compared to other 2’-ribose modifications [51,54]. Moreover, combination of phosphorothioate and 2’-F modifications have exhibited toxic effects in siRNAs [51]. The advancement of RNA-based therapeutics requires optimizations in designing the positional modifications for siRNA testing to achieve the maximum *in vivo* activity.

**Figure 3.**
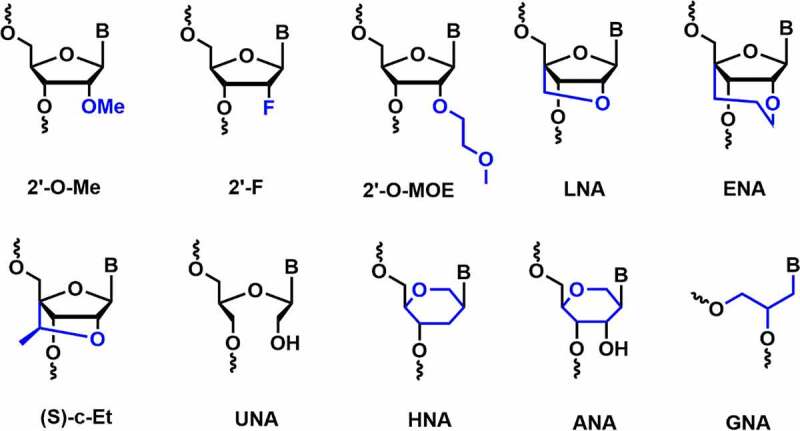
Various C2’ and non-canonical sugar chemical modifications.

Other effective sugar chemical modifications are also tested for RNAi activity including, locked nucleic acids (LNA), anhydrohexitol nucleic acids (HNA), altritol nucleic acids (ANA), ethylene-bridged nucleic acids (ENA), constrained ethyl nucleic acids (cEt), unlocked nucleic acids (UNA) and glycol nucleic acids (GNA) **[**Figure 3**]**.

LNA is known to impart an impressive duplex stabilization of 2–8°C per modification [51]. It is well tolerated in the passenger strand, whereas the incorporation at the first position of the 5’-end of the duplex completely abolishes the RNAi activity [51,55]. Furthermore, LNA modification improves nuclease resistance and reduces innate immune responses [51,55]. Ethylene-bridged nucleic acid (ENA) is a homolog of LNA which has an exclusive *North* sugar conformation and has better AON properties than LNA. ENA exhibits an increase in *T_m_* by 5.2°C per modification compared to RNA. It is also more resistant to nucleases than DNA or LNA [56]. The 2’,4’-constrained 2’-*O*-ethyl modifications (cEt) consist of an exocyclic methyl group, affords *N*-type sugar pucker and has high nuclease resistance without affecting binding affinity towards RNA. The thermal stability of (*R*)- and (*S*)-cEt oligomers with RNA complement showed a *T_m_* value of ~ 4.7°C and ~ 4.5°C per modifications [57].

Unlocked nucleic acids (UNA) and glycolic nucleic acid (GNA) are destabilizing modifications that reduce thermal stability by around 5–8°C and 5–18°C per modification in RNA duplexes, respectively [51,58,59]. Incorporation of a single UNA modification in both the strands of siRNA showed improved RNAi activity compared to the unmodified siRNAs [60]. *In vivo* studies in mice models showed their prolonged biostability compared to the LNA-modified siRNA [58]. Moreover, UNA modification can reduce off-target effects when incorporated in the seed region of the guide strand [61]. Likewise, the incorporation of a single GNA modification in the seed region of the guide strand mitigated off-target activity in rat hepatocytes. The reduction in off-target effects in rat hepatocytes helps to minimize the hepatotoxicity and improves the safety profile of siRNA *in vivo* [62].

Anhydrohexitol nucleic acids (HNA) and altritol nucleic acids (ANA) modifications employed in siRNA involve pyranose rings instead of furanose. HNA and ANA form antiparallel W-C base pairing with the complementary RNA strands, which helps in the stabilization of HNA/RNA and ANA/RNA duplexes [63]. Fisher *et al*. reported HNA and ANA modified siRNAs targeting the human *B-Raf* gene in human melanoma cells (A375 cells) [64]. Both the modifications were tolerated at the 3’-end of passenger strand but showed decreased potency when incorporated at the 5’-end of the guide strand [64].

Manoharan and co-workers investigated RNAi activity using ANA modification at each position in both the strands of siRNA targeted against TTR mRNA in mouse hepatocytes [65]. Incorporation of ANA modification was well tolerated at positions 6 and 7 in the seed region of the guide strand but is detrimental at the 5’-end of the guide strand. As previously reported by Fisher *et al*., the reduction in RNAi activity at the 5’-terminus of the guide strand could be due to the lack of phosphorylation of 5’-ANA nucleotides by endogenous intracellular kinases. Further analyses suggested that ANA modified siRNA does not influence off-target activity. This could be because the ANA does not fall under the destabilizing modification category [65].

After successful implementation of 2’-sugar modifications in the siRNA drugs, many other modified sugar scaffolds such as bridged nucleic acids (BNA), pyranose ring containing nucleic acids, and acyclic nucleic acids containing oligonucleotides were synthesized and tested for RNAi activity [10,11]. Importantly, most of the ribose modifications in siRNAs help to reduce the off-target effects and innate immune stimulation, improve metabolic stability and specificity, and nuclease resistance.

## 4. Overview of dual modifications

C-4’ modification is another important position in the ribose sugar, which controls the properties of nucleic acids without affecting the W-C base pairing in siRNA duplexes [66]. During the last decade, various dual-sugar chemical modifications have been employed in siRNAs. Incorporation of 4’-modification could potentially include the additional stereoelectronic effects, which subsequently alter the sugar conformation^67^,68]. Incorporation of 2’,4’-modifications in nucleoside increase in nuclease stability and RNAi compatibility. We have critically discussed various 2’,4’- and 2’,5’- sugar modifications, *E/Z-*vinyl phosphonate modification, and northern methanocarbacyclic (NMC) modifications. Interestingly, these modifications strongly influence the pharmacokinetic and pharmacodynamic properties of siRNA duplexes [66-[69]^69^. Moreover, they show increased nuclease resistance, prolonged gene silencing activity, high tissue accumulation, and reduced off-target effects. In the following subsections, we aim to cover all dual sugar modifications reported so far and to discuss their effect on siRNA stability and RNAi activity.

### 4.1. 4’-thio-C2’ modifications

The essential criteria for designing the chemically modified oligonucleotides are to enhance the thermodynamic and nuclease stability for their therapeutic applications. Matsuda and co-workers developed 4’-thioribonucleosides and tested their serum stabilities and RNAi activities [70]. Incorporation of four 4’-thioribonucleosides modifications at both ends of the passenger strand and at 3’-end of the guide strand targeting *luciferase* genes showed a potent RNAi activity in several cell lines, including NIH/3T3, HeLa, and MIA PaCa-2 [71]. The modification is well tolerated in the passenger strand rather than guide strand. Moreover, comparative study showed that 4’-thioribonucleosides modification is compatible with the RNAi machinery and improved long-term silencing effect compared to 2’-*O*-Me nucleosides.

**Figure 4.**
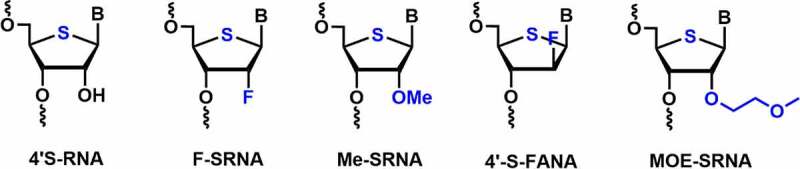
Various 4’-thio-*C*2’ modifications.

To check the synergistic effect of 4’-thio RNA (SRNA) with 2’-modifications, 2’-F-4’-thio RNA (F-SRNA), and 2’-*O*-Me-4’-thio RNA (Me-SRNA) building blocks were synthesized and incorporated into the siRNAs. 4’-thio modification (4’-S) in a siRNA duplex significantly enhanced the nuclease stability **[**Figure 4**]** [71]. Me-SRNA showed the highest nuclease resistance against endonucleases and exonucleases as well as the highest half-life stability in 50% human plasma as compared to F-SRNA, 2’-*O*-Me RNA, and 2’-fluoro RNA modifications 71.

Me-SRNA exhibited an additive effect of MeRNA and SRNA which was evident in its higher nuclease stability (>24 h) among all the modifications used in the study. The nuclease resistance rank order was observed as Me-SRNA>F-SRNA>SRNA>MeRNA, FRNA, RNA, and DNA [71]. In contrast, melting temperature (*T_m_*) was highest for F-SRNA in RNA duplex compared to Me-SRNA, 2’-fluoro, and 2’-*O*-Me modified RNAs. F-SRNA has shown synergistic improvement in *T_m_* value [71].

In another study, gene silencing activity was evaluated using Me-SRNA modified siRNA in HeLa cells. The incorporation of three consecutive Me-SRNA modifications at both ends of the passenger strand showed optimum anti-luciferase activity (79%) in HeLa-Luc cells [72]. Moreover, Me-SRNA modified siRNA has shown prolonged gene silencing activity (after 3 days) due to its remarkable nuclease resistance. These results indicated that the nuclease stability is an important factor in determining gene silencing efficiency.

4’-S-FANA modification in the siRNA duplex showed a slight thermal destabilization of ~ 1–1.4°C per modification [73]. Incorporation of one or two 4ʹS-FANA modifications in either of the strands showed potent RNAi activity against the firefly luciferase gene [73]. Saito *et al*. reported the synthesis, hybridization, and nuclease activity of 4’-*S*-2’-MOE (MOE-SRNA) modified RNAs [74]. In hybridization studies, MOE-SRNA has exhibited *T_m_* value ~3.4°C per modification against complementary RNA. Nuclease stability studies using Me-SRNA, 2’-*O*-MOE, 2’-*O*-Me, 4’-thio RNAs in comparison with MOE-SRNA modified RNAs were performed in human serum. The synergistic effect in MOE-SRNA showed the highest nuclease resistance (*T_1/2_* greater than 48 h) among all modifications used in the study [74].

Overall, the incorporation of 4’-thioribonucleosides along with the C2’-modification (2’-OMe, 2’-F, 2’-MOE) in siRNAs resulted in greater nuclease resistance and improved gene silencing activity than the 2’-modifications alone. However, detailed *in vivo* studies are needed to be performed using animal models in order to prove the safety and efficacy of these dual sugar chemical modifications for clinical purposes.

### 4.2. 4’/5’-aminoalkyl/C2’ modifications

**Figure 5.**
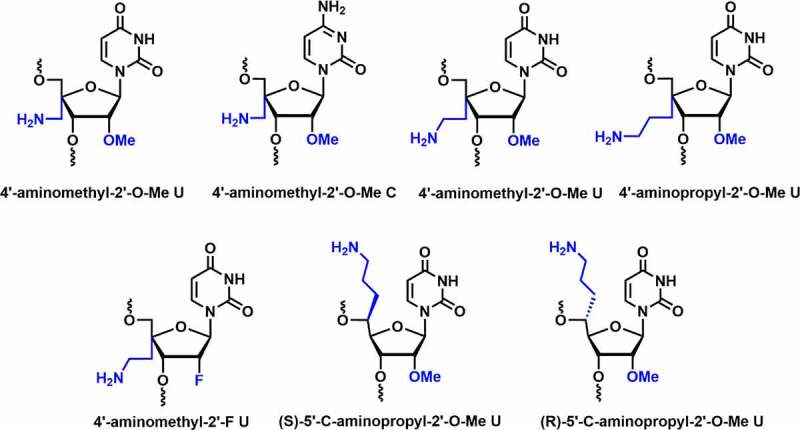
Various 4’/5’-aminoalkyl-C2’ modifications.

Since 4’-modification is in the proximity of phosphodiester linkage, it was predicted that the 4’-modification could be effective in enhancing the nuclease resistance. In 2012, Gore *et al*. reported the synthesis of 4′-aminomethyl-2′-*O*-Me uridine and cytidine modified siRNAs and evaluated their nuclease stability and gene silencing activity **[**Figure 5**]** [75]. The 4′-aminomethyl-2′-*O*-Me (4’-AM-2’-*O*-Me) modification displayed C2′-*endo* (*S*-type) conformation. Compared to unmodified duplexes, modified siRNA showed thermal destabilization of ~1°C per modification.

Modifications were well tolerated at various positions in the passenger strand but decreased in RNAi activity at position 3 in the seed region of the guide strand. Serum studies indicated that siRNA with modification at position 3 in the seed region and at the overhang remain intact up to 8 hours in human serum [75]. Ueno and co-workers reported the synthesis of 4′-aminoethyl-2′-*O*-Me-U and 4′-aminopropyl-2′-*O*-Me-U and investigated the influence of 4’-*C*-aminoalkyl on serum stability, thermal stability, and RNAi activity **[**Figure 5**]** [76]. Both modifications adopt the C2′-*endo* sugar conformation similar to 4’-AM-2’-*O*-Me modification. The incorporation of 4′-aminoethyl-2′-*O*-Me-U and 4′-aminopropyl-2′-*O*-Me-U modifications decreased the thermal stability of the siRNA duplex by ~1-2°C per modification. Longer side chains could result in more unfavourable entropy, implying that the 4′-*C*-aminoalkyl modification can affect RNA duplex stability due to altered sugar conformation.

Incorporation of 4’-aminopropyl group modified siRNA at position 11 from the 5′-end showed a slight reduction in RNAi activity, suggesting that the 4′-aminopropyl modification near the cleavage site has minor impact on Ago2 recognition [76]. Incorporation of 4′-aminoethyl-2′-*O*-Me-U and 4′-aminopropyl-2′-*O*-Me-U at position 2 inhibits RNAi activity, whereas at position 8 from the 5’-end of the guide strand does not affect the RNAi activity. It was further discovered that 4′-aminoethyl-2′-*O*-Me-U and 4′-aminopropyl-2′-*O*-Me-U modified siRNAs remain intact in bovine serum for 6 h. Overall, the siRNAs were well tolerated in the passenger and guide strands except in the seed region [76].

Ueno and co-workers also reported the synthesis of 4’-aminoethyl-2’-deoxy-2’-F-U modified siRNAs and tested their thermal stability, serum stability, and RNAi activity [77]. The modified sugar adopts a C3’-*endo* sugar conformation due to the presence of 2’-F substituent instead of 2’-*O*-Me. It indicates that the 2’-F group increases duplex stability. It could be due to the stronger gauche effect and C-H–F hydrogen bond that stabilizes the 4’-aminoethyl-2’-F U modification. The serum stability was tested in bovine serum using siRNAs containing 13 modifications of 4′-aminoethyl-2′-*O*-Me-U and 4’-aminoethyl-2’-F-U in their strands. The results revealed that 55% and 48% of the modified siRNAs remained intact after 48 h of incubation, respectively [77].

Incorporating 4’-aminoethyl-2’-F modification at position 11 (near the cleavage position) from the 5’ end of the guide strand in the siRNA resulted in higher RNAi activity than the 4’-aminoethyl-2’-*O*-Me modified siRNA. Gene silencing results showed that the 4’-aminoethyl-2’-F modification is more suitable at the cleavage site compared to the 4’-aminoethyl-2’-*O*-Me modification. Overall, the results indicated that 4’-aminoethyl-2’-F modification increases the thermal stability, RNAi activity, and reasonable nuclease stability compared to the 4’-aminoethyl-2’-*O*-Me modification. Thus, the 4’-aminoethyl-2’-F modification could be further tested in siRNAs for the development of therapeutic siRNA molecules [77].

The successful incorporation of aminoalkyl modification at the C4’-position in the sugar and encouraging results open the door for further investigations. Ueno and co-workers synthesized (*R*), (*S*)-isomers of 5′-*C*-aminopropyl-2′-*O*-Me U and compared their properties with 4′-*C-*aminopropyl-2′-*O*-Me modification **[**Figure 5**]** [78]. The incorporation of (*S*)- and (*R*)-isomer decreased the thermal stability of dsRNA by ~1.0 and ~2.7°C per modification, respectively. The destabilizing effect in (*R*)-isomer may be due to the interference with hydration around the phosphate backbone. Both isomers adopt C3′-*endo* sugar conformation, whereas 4′-*C-*aminopropyl modification exhibits C2’-*endo* conformation. In a dual-luciferase reporter assay, RNAi activity was tested in HeLa cells using modified and unmodified siRNAs. Incorporating a single modification in the passenger strand was well-tolerated and effectively suppressed *Renilla* luciferase gene expression close to control siRNA. Incorporation of (*R*)-isomer at position 8 from the 5’-end of the guide strand reduced RNAi activity. Serum stability was evaluated using (*R*), (*S*)-isomers modified siRNAs in bovine serum, both isomers containing siRNAs remained intact for up to 6 hours of incubation. In conclusion, the (*S*)-isomer was effective in increasing both the thermal and serum stability of siRNA duplexes compared to the (*R*)-isomer and 4′-*C-*aminopropyl-2′-*O*-Me modified siRNA [78]. These results suggest that the (*S*)-isomers of 5′-*C*-aminopropyl-2′-*O*-Me U could be the most suitable candidate for future siRNA therapeutic interventions.

In summary, these investigations suggest that the close proximity of 4’-aminoalkyl and 5’-aminoalkyl groups to neighbouring phosphate significantly increased nuclease resistance and compatibility with RNAi machinery. However, a detailed comparative studies using these modifications will help to find the most suitable modifications for siRNA therapeutics.

### 4.3. C4’-Guanidino-C2’-modifications

**Figure 6.**
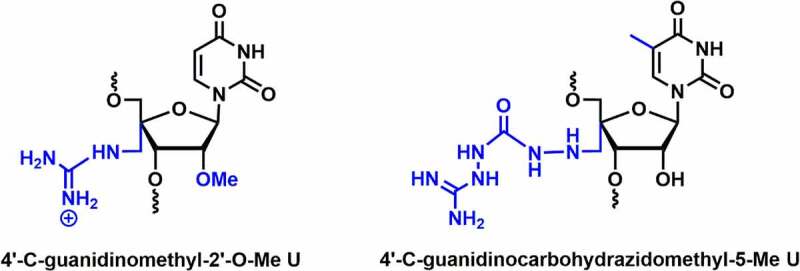
C4’-Guanidino containing 2’ modifications.

Ueno and co-workers synthesized the 4’-*C*-guanidinomethyl-2’-*O*-Me and 4′-aminomethyl-2′-*O*-Me uridine modified siRNAs and investigated their cell permeability and RNAi activity **[**Figure 6**]** [79]. The 4’-*C*-guanidinomethyl-2’-*O*-Me modified dsRNA caused a reduction in *T_m_* by ~3.3°C per modification. 4’-*C*-guanidinomethyl-2’-*O*-Me and 4′-aminomethyl-2′-*O*-Me modifications are well tolerated in the passenger strand, inhibit the expression of the *renilla luciferase* gene similar to the unmodified siRNA. This modified RNA was stable up to 12 h in bovine serum. Cell membrane permeability was tested using fluorescent-labeled RNA in HeLa cells. RNA containing 4’-*C*-guanidinomethyl-2’-*O*-Me modification showed higher fluorescent intensity inside the HeLa cells compared to RNA containing 4’-aminomethyl modification. Overall, this finding suggests that the 4’-guanidinomethyl modification is a better modification for increasing serum stability and cell permeability than the 4’-aminomethyl modification [79].

4′-*C*-guanidinocarbohydrazidomethyl-5-methyl uridine (GMU) modified siRNAs were synthesized and evaluated for their gene silencing studies against STAT3 mRNA **[**Figure 6**]** [80]. The GMU modification tunes the sugar conformation towards the C3′-*endo*, which is necessary for A-type RNA integrity. The melting temperature (*T_m_*) of the duplex increased by 2.6–2.9°C at the overhang position of either of the strands. *In silico* molecular modelling studies showed that the *C*4’-guanidinium linkers confer additional non-covalent interactions, which impart thermodynamic stability in the RNA duplex [80]. Further, gene silencing results suggested that a couple of 4’-guanidinium modifications in the overhang positions were well tolerated in both passenger and guide strands and could achieve comparable knockdown efficiency to unmodified siRNAs. siRNA duplex containing dual GMU modifications in overhang positions of both the strands remained completely intact up to 12 h in human serum, whereas 50% remained intact after 72 h in same condition [80].

Overall, guanidinium modification at the C4’-position enhances nuclease resistance and cell membrane permeability as compared to the 4’-aminomethyl-2’-OMe modification. These findings suggest that guanidinium modifications are well tolerated in the passenger and overhang positions. In future, the guanidinium modifications should be investigated more deeply to explore the therapeutic properties of siRNA.

### 4.4. C4’-O-Me/C2’-modifications

Most chemical modifications at the C-2’ position is reported to improve the thermodynamic stability, nuclease resistance and allow for favourable interactions with RISC protein assembly. In contrast, substitution at the 4’-position alters the sugar conformation and minimizes nuclease degradation due to sterically crowding around two vicinal phosphodiester linkages.

**Figure 7.**
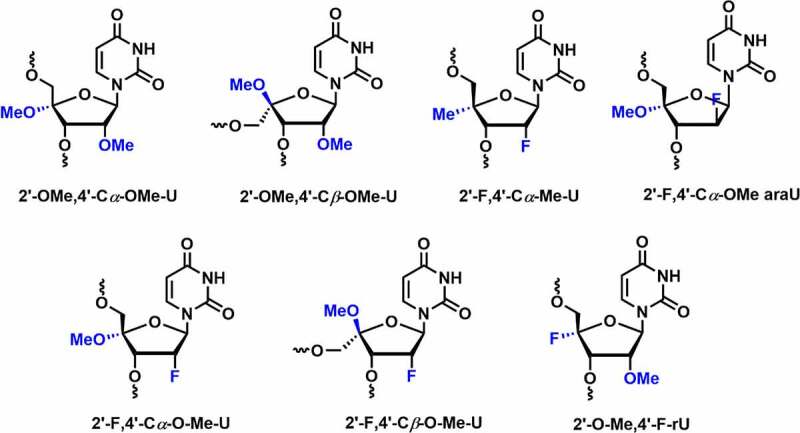
Various C4’-*O*Me/F/Me-C2ʹmodifications.

In 2017, Damha and co-workers reported the synthesis and conformational analysis of 4′-*C*-OMe-2′-deoxy-2′-F-U analogs containing both the alpha (*C*4′-*α*) and beta (*C*4′-*β*) epimers **[**Figure 7**]** [81]. Thermal stability data indicate that the introduction of the C4’-*α-*OMe modification in RNA duplex does not have any influence on the duplex stability *(∆T_m_* = 0°C). However, C4’-*β-*OMe led to thermal destabilization by ~9°C when incorporated into the dsRNA duplex. The C4’-*α-*OMe epimer imparts anomeric (non-bonding O4′→ antibonding σ* C4′O) and hyperconjugation effects, which tune the sugar into the C3’-*endo* (*North*) conformation. In the case of the C4’-*β-*OMe epimer, a strong gauche effect due to C2’-fluoro favours the *North* conformation, while the anomeric effect of C4’-*β-*OMe epimer stabilizes the *South* conformation. These two stereoelectronic effects result in a ratio of (2:5) *N/S* conformational preferences [81].

RNAs with 2’-F modification exhibits limited enzymatic stability [51]. It is interesting to evaluate metabolic stability by introducing 4′-*C*-OMe-2′-deoxy-2′-F-U into oligonucleotides. Serum stability results showed that 2’-F modification in the oligonucleotides shows complete degradation in less than 1 hour. Incorporation of 2′-F-4′-*C*α-OMe U showed enzymatic stability against snake venom phosphodiesterase (SVPD) for almost 1 h, whereas with C*β*-epimer no such degradation was observed within 24 h.

RNAi activity was tested and compared within both the epimers and mono modifications, including 2′-*O*-Me, 2′-F, and PS. Modified siRNAs were targeted against transthyretin (*TTR*) or firefly luciferase (*Luc*) mRNA. Incorporation of the 4′-C*α-O*-Me epimer was well tolerated at position 11 in the passenger strand. In contrast, silencing activity was dropped at position 2 in the guide strand; it could be due to the steric clashes with the RISC protein assembly. The introduction of three 4′-C*α*-OMe modifications at positions 4, 18, and 20 in the guide strand exhibited a minimal reduction in RNAi activity. Interestingly, the incorporation of 4′-C*β*-OMe at position 2 in the guide strand showed a 48-fold loss in activity [81]. Overall, 4′-C*β*-OMe-2’-F shows the higher nuclease resistance as compared to the 2’-F and 4′-C*α-O*-Me epimers. Comparatively, it exhibited lower thermal stability and less compatibility with the RNAi machinery. These results suggest that 4′-C*α-O*-Me epimer could be a suitable candidate for evaluation of future siRNA therapeutics.

In 2018, Egli and co-workers investigated the potential effects of fluoro and methoxy modifications at C2’ and C4’ positions in siRNAs [82]. The dual C2’/C4’ modifications including 2’-F, 4’-C*α*-OMe-U (*Ufo*), 2’-F,4’-C*β*-OMe-U (*Ufob*), 2’-OMe,4’-C*α*-OMe-U (*Uo*), 2’-OMe, 4’-C*β*-OMe-U (*Uob*), and 2’-F,4’-C*α*-Me-U (*Ufme*) were synthesized and evaluated their potential effects on RNAi machinery **[**Figure 7**]** [82]. All three C*α-*epimers i.e. *Ufme, Ufo*, and *uo* preferentially adopt the C3’-*endo* (*N*-type) conformation. On the other hand, crystallographic data demonstrated that all the C*β-*epimers such as *Ufob* and *Uob* had an *N*-type C2’-exo sugar conformation. Thermal melting studies suggested that C*α-*epimers adopting *North* conformation exhibited comparable *T_m_* values as unmodified RNA duplex. The C*β-*epimers (*Ufob* and *Uob*) displayed greater destabilization in the RNA duplex up to 9°C per modification.

The 2’-F and 2’-*O*-Me modified oligonucleotides, as expected, were degraded within 1 h of incubation time in SVPD. Incorporation of C4’-substituents that are C*α-*epimers (*Ufme, Ufo*, and *uo)* enhances serum stability as compared to the 2’ modifications. The C*β-*epimers (*Ufob* and *Uob*) shows more than 90% full-length product after 24 h of incubation in SVPD. *In vitro* gene silencing activity was evaluated by incorporating the C*α- and Cβ-*epimer residues in various positions of siRNAs targeting the transthyretin (*TTR*) mRNA in mouse hepatocytes. Gene silencing results were found to be similar as discussed above [81]. On the contrary, *Ufme* modification is well tolerated when incorporated at position 2 in the guide strand and at position 11 in the passenger strand.

The crystal structures of *Ufo* and *Ufob* modified 8-mer RNAs bound to human Ago2 showed that the steric conflict arises between Ago-2 amino acids and RNA residues due to the presence of the C4’-*O*-Me group in both *Ufo* and *Ufob* nucleotides [82]. Overall, the results suggested that the C*α-*epimers showed enhanced serum stability, thermal stability and were compatible with the RNAi machinery. On the other hand, *Cβ-*epimers are found to be destabilizing modifications with greater nuclease resistance.

In 2019, Damha and co-workers compared the gene silencing efficiency and thermal stability by incorporating various chemical modifications including 2’-F, 2’-araF, 2’-*O*-Me, 2’-F,4’-*O*-Me, 2’-araF,4’-*O*-Me, 2ʹ4’-di-*O*-Me, and 2’-*O*-Me,4’-F into the internal or overhang positions of the guide strand in siRNA duplex **[**Figure 7**]** [83]. The modifications were incorporated at internal positions 6, 13, 14 as well as overhang positions 20 and 21 of the guide strand. Thermal stability results showed increased duplex stability (Δ*T_m_ *= +0.3°C to 3.0°C) for all these modifications at both the combined positions.

*In vitro* studies were carried out in HeLa cells using siRNAs targeting the *firefly luciferase* gene. siRNAs containing 2’-*O*-Me,4’-F, 2’-F,4’-*O*-Me, and 2’-*O*-Me modifications at the internal positions (6, 13, and 14) conferred excellent antiluciferase activity, whereas 2’-araF, 4’-*O*-Me, and 2ʹ4’-di-*O*-Me decreased RNAi activity. siRNAs containing 2’-F,4’-*O*-Me, 2’-araF,4’-*O*-Me, and 2ʹ4’-di-*O*-Me modifications at overhang positions (20 and 21) offered excellent antiluciferase activity. Gene silencing activity was excellent for siRNAs containing 2’-F or 2’-*O*-Me (internal positions 6, 13, and 14) in combination with the 2’-F,4’-*O*-Me, 2’-araF,4’-*O*-Me, and 2ʹ4’-di-*O*-Me modifications at overhang positions (20, 21). This study indicates that dual 2ʹ4’-modifications show potent RNAi activity when incorporated at the overhang position [83]. In summary, these studies suggest that 4’-*O*-Me modification with 2’-F is most compatible with the RNAi machinery and enhances nuclease stability significantly. However, further *in vivo* evaluation in animal models is required to optimize the properties associated with the RNAi therapeutics.

### 4.5. 5’-(E/Z)-vinylphosphonate-C2’ modification

**Figure 8.**
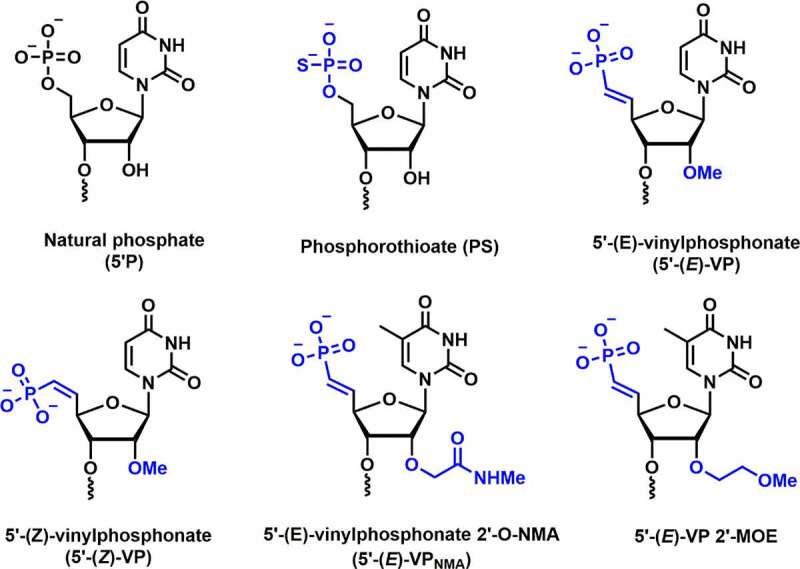
5’-(*E/Z*)-Vinylphosphonate-C2’ modification.

Standard siRNA comprises a fundamental RNA structure having phosphodiester linkage between the 3’ and 5’ of the sugar moieties. Replacement of the phosphodiester linkages with phosphorothioate (PS), boranophosphate (BP), and other similar linkages have been explored to design and develop new oligonucleotides with improved RNAi activity **[**Figure 8**]**. Phosphorothioate modifications protect siRNA from 3’-exonucleases *in vitro* and *in vivo* [51]. It was found that more than 50% PS content in the siRNA duplex causes toxicity *in vitro* and *in vivo* [51,84]. Incorporation of PS or BP modifications in the central region of the guide strand inhibits RNAi activity, which indicates that the central position in the guide strand is sensitive to backbone modifications [51,85]. Moreover, PS modification increases cellular uptake of siRNAs in the absence of transfection agents. This is due to the nonspecific binding of PS with cell membrane receptors that promote endocytosis of the siRNA [86].

Introduction of vinylphosphonate (VP) backbone modification in the 5’-end of guide strand of siRNA is known to improve tissue concentration, RISC loading, duration of silencing, and metabolic stability. VP-modified siRNA is also recognized as a phosphate mimic in RISC AGO-2 assembly. Jadhav and co-workers evaluated the efficacy of 5’-VP modification *in vitro* and *in vivo* [87]. The dephosphorylation efficiency was tested by incubating the unmodified and 5’-VP-modified siRNA conjugates in the rat liver tritosomes. The lysosomal enzymes present in tritosomes are known for their 5’-dephosphorylation activity. The authors observed no loss of vinyl phosphonate group after prolonged incubation inferring that vinylphosphonate is metabolically stable phosphate modification.

*In vitro* potency was tested using VP-modified siRNAs in mouse hepatocytes, which resulted in greater potency compared to unmodified siRNAs. *In vivo* studies were carried out in C57BL/6 female mice to evaluate *ApoB* knockdown. Incorporation of 5’-VP modification in the guide strand showed a 40% decrease in LDL level *in vivo*. In comparison to natural phosphate (5’-P), these 5’-VP alterations result in up to a 20-fold increase in *in vitro* potency in the primary mouse hepatocytes and up to a 3-fold increase in *in vivo* activity [87]. Overall, these findings suggest that the VP modification at position 1 could be an excellent alternative and will not heavily rely on 5’-phosphorylation for better *in vivo* silencing efficiency. Furthermore, 5’-VP tends to improve the metabolic stability of such conjugates, resulting in an increased potency *in vivo* [87].

Joshua-tor and co-workers synthesized 5’-*E*-vinylphosphonate-2’-*O*-methyl-uridine (5’-*E*-VPu) modified guide strand targeting TTR RNA and determined the crystal structure of the hAgo-2 protein bound to guide strand containing VP modification [Figure 8] [88]. Crystallographic studies revealed that replacing the 5’-phosphate by 5’-*E*-VPu does not interrupt binding interactions between hAgo-2 and guide strand; rather it induces favourable interactions with Ago-2 binding pockets over 5’-phosphate. *In vitro* studies were carried out using 5’-*E*-VPu-TTR siRNA-GalNAc conjugates in primary mouse hepatocytes. Results revealed that the 5’-*E*-VPu-TTR modified siRNA was ~2-fold more efficient compared to 5’-OH-TTR siRNA (control).

*In vivo* activity was studied using GalNAc-siRNA conjugate having 5’-*E*-VPu modification in the guide strand to downregulate the level of TTR mRNA in mice. Subcutaneous administration of the 5’-*E*-VPu-TTR siRNA revealed 85% silencing of the TTR mRNA on day 7 post-dose at 1 mg/kg dose, whereas only 64% gene silencing was observed using 5’-OH-TTR siRNA. The results imply the improved incorporation of 5’-vinylphosphonate bearing siRNA into hAGO-2, *in vivo* metabolic stability of VP, and prolonged stability of RISC complex *in vivo* could be responsible for the persistent silencing activity using 5’-VP modifications [88].

Rajeev and co-workers reported the siRNA activity *in vitro* and *in vivo* using (*E)* and (*Z)* isomers of 5’-VP uridine in combination with 2’-bulky substituent 2′-*O*-[2-(methylamino)-2-oxoethyl] (5’-VP_NMA_) [Figure 8] [89]. These 5’-(*E*)-VPu and 5’-(*Z*)-VPu modified siRNA conjugates were used to target TTR and ApoB mRNA in mouse hepatocytes *in vitro*. When compared to the control siRNA conjugate, the 5’-(*E*)-VPu modified siRNA demonstrated a 2-fold efficacy. On the contrary, 5’-(*Z*)-VPu has shown poor silencing activity. A similar trend of activity was observed against ApoB mRNA. Surprisingly, the bulkier 5′-(*E*)-VP_NMA_ modification in the guide strand demonstrated a 15-fold increase in potency when compared to the parental siRNA. AGO-2 loading data suggested that 5’-(*E*)-VPu has higher AGO-2 loading than the parent siRNA and 5’-(*Z*)-VPu siRNA. In addition, *in vivo* potency was investigated using these siRNA conjugates by targeting the TTR and ApoB mRNA in mice [89].

*In vivo* studies showed an 85% reduction of TTR mRNA using 5’-(*E*)-VPu modified siRNA at day 7 post-dose compared to parent conjugate ~64%). In contrast, 5’-(*Z*)-VPu showed reduced *TTR* silencing (~40%) which is inferior to parent and *E* isomer siRNAs. The 5’-(*E*)-VPu and 5’-(*Z*)-VPu modified siRNA conjugates reduces the level of *ApoB* gene in mice by 62% and 28%, respectively. Overall, these results showed improved *in vivo* activity using both the (*E*)-VPu conjugates targeting *ApoB* and *TTR* gene. This could be due to the improved loading of the guide strand containing (*E*)-VPu modification. Further, *in vivo* siRNA potency was evaluated using 2’-*O*-NMA alone and 5’-(*E*)-VP_NMA_ targeting the *ApoB* gene and was compared to 5’-(*E*)-VP modification.

The 2’-*O*-NMA showed poor activity suggesting that 5’ phosphorylation is not compatible with the bulkier 2’-substituent. The 5’-(*E*)-VP_NMA_ showed superior *ApoB* silencing activity than 5’-(*E*)-VP, suggesting that the 2’-NMA group is accommodated in the RISC complex without any steric hindrance. Crystal structure of Ago-2 bound to 2’-NMA-T-modified guide strand revealed that NMA mediates stabilizing interactions with the guide and/or Ago-2 protein residues [89].

Prakash and co-workers demonstrated synergistic effects by combining PS and VP backbone modifications on fully 2’-modified (2’-F, 2’-*O*-Me, and 2’-*O*-MOE) siRNA-GalNAc cluster conjugates targeting PTEN mRNA [90]. Their *in vitro* results in HeLa cells showed greater potency compared to the unconjugated PS modified siRNAs. Similarly, *in vivo* data in mice showed that the GalNAc cluster conjugated siRNAs containing a combination of PS and 5’-VP has considerably higher potency (5–10-fold) than unconjugated PS modified siRNA with 5’-natural phosphate. These results suggest that the 5’-VP modification is metabolically stable and eliminates the need for re-phosphorylation of guide strands prior to loading into the RISC complex [90].

Khvorova and co-workers investigated the chemical phosphorylation efficiency, serum stability, *in vitro* and *in vivo* silencing activity using siRNA-cholesterol conjugates (hydrophobic siRNA/hsiRNA) containing a 5’-(*E*)-VP modification [91]. Metabolic stability studies suggested that the 5’-phosphate hsiRNA undergoes rapid dephosphorylation compared to the 5’-(*E*)-VP modified hsiRNA. *In vivo* efficacy was compared using the 5’-hydroxy, 5’-phosphate, and 5’-(*E*)-VP hsiRNAs duplexes, which were injected subcutaneously and intravenously targeting *Ppib* mRNA in mice. The results confirmed higher potency for 5’-(*E*)-VP (66% subcutaneous and 57% intravenous administration) compared to the 5’-OH and 5’-P hsiRNAs (30–50% subcutaneous and 25–30% intravenous administration).

Moreover, tissue accumulation for 5’-(*E*)-VP hsiRNAs is greater than the 5’-P hsiRNAs which could also explain the higher *in vivo* potency. The increased potency and tissue accumulation of 5’-(*E*)-VP-hsiRNA explain their resistance to phosphatase and exoribonucleases. To confirm these results, 5’-(*E*)-VP-hsiRNA, 2’-fully modified guide strand with and without PS modification were incubated with 5’-to-3’-exoribonuclease, XRN1 [91]. The findings illustrate that the VP modification alone could suffice to protect the guide strand from the 5’-to-3’-exoribonuclease.

In conclusion, VP modification is quite well explored in the field of siRNA therapeutics, which may lead to future clinical implications. 5’-(*E*)-VP modification is an alternative to PS backbone modification at position 1 and protects the guide strand from nucleases. *In vitro* and *in vivo* data revealed that the 5’-(*E*)-VP modification has improved tissue accumulation, RISC loading, prolonged gene silencing, and metabolic stability. In addition, crystallographic studies revealed that the 5’-(*E*)-VP modification along with the guide strand perfectly fits into the hAgo-2 protein and showed favourable interactions in hAgo-2 assembly.

### 4.6. 2’,5’ dual modifications in single-stranded siRNA (ss-siRNA)

**Figure 9.**
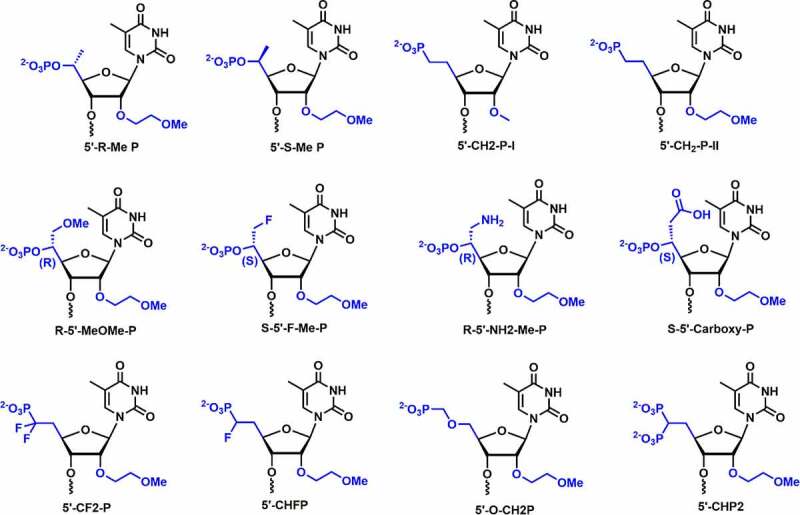
Various 2’,5’ dual modifications.

In 2012, Lima *et al*. studied the *in vivo* potency of chemically modified single-stranded siRNA (ss-siRNA) including the PS modification and found that the ss-siRNA do not show any activity [92]. The finding indicated that 5’-phosphate in ss-siRNA undergoes dephosphorylation rapidly *in vivo* which is primarily required for silencing activity. Prakash and co-workers introduced various 5’-structural analogs at position 1 in ss-siRNAs and compared their silencing activities. 5’-modifications including 5’-Me, 5’-Me-OMe, 5’-fluoromethyl, 5’-aminomethyl, 5’-carboxymethyl, *α*-fluoromethylenephosphonate (5’-CHF-P), *α,α*-difluoromethylenephosphonate (5’-CF_2_-P), 5’-methylenephosphonate, 5’-(diethylphosphonate), 5’-bisphosphonate, etc. were incorporated at position 1 of ss-siRNA targeting to PTEN mRNA for testing *in vitro* activity [93] **[**Figure 9**]**. 5’-(*R*)-Me substituent in ss-siRNA showed improved potency (3-5-fold) over parental and *S*-isomer

Most of the 5’-substituents incorporated at position 1 in ss-siRNAs have shown lower potencies relative to parental ss-siRNAs. This demonstrated that varying the charge density, steric crowding, and orientation of 5’-phosphate have significant effect on the potency of ss-siRNAs. Introduction of 5’-(*E*)-VP substituents showed similar potency to parental ss-siRNAs, whereas, 5’-(*Z*)-VP exhibited lower silencing activity compared to parental ss-siRNA [93]. These findings indicated that the rigid conformation of 5’-(*E*)-VP structurally resembles a phosphate group and is accommodated in the Ago-2 binding pocket. 5’-(*E*)-VP could be a metabolically stable phosphate mimic for 5’-phosphate in ss-siRNA therapeutics.

### 4.7. 2’-fluoro-Northern-Methanocarbacyclic modification

**Figure 10.**
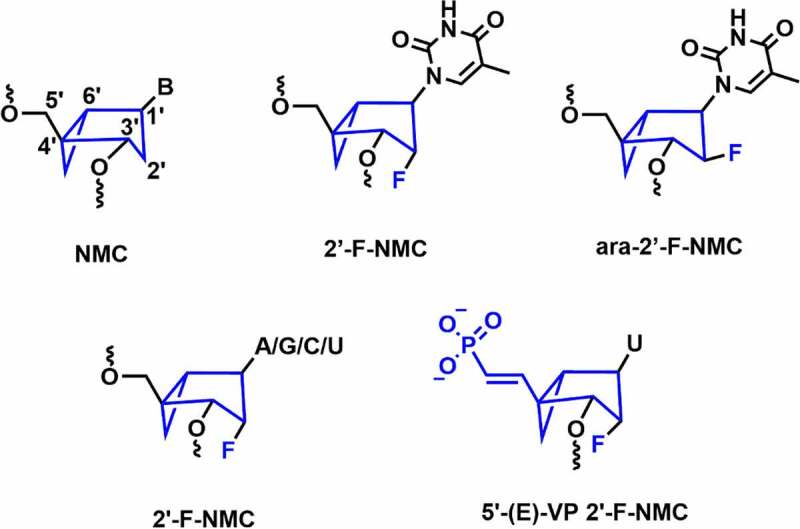
Northern methanocarbacyclic and its fluorinated analogues.

In 1994, Altmann and co-workers reported the northern methanocarbacyclic (NMC) nucleosides as a structural mimic for C3’-*endo* sugar [94] It is a carbocyclic bicyclo[3.1.0]hexane system and adopts pseudo boat C2’-*exo* (*North*) conformation which is a mimic of the C3’-*endo* conformation due to the methylene bridge between the *C*4’ and *C*6’ positions **[**Figure 10**]** [95,96]. Interestingly, the methylene group locks the conformation towards the *North* conformation without any need for an electron-withdrawing group at 2’-position. In 2014, Jung and co-workers reported the synthesis of 2′-F-NMC thymidine and ara-2′-F-NMC thymidine analogues and studied the duplex stabilizing properties **[**Figure 10**]** [95]. The incorporation of a single F-NMC nucleotide showed thermal stabilization of the duplex (+ 2.2°C per modification). The 2’-F-NMC modification shows high affinity towards RNA as compared to 2’-F nucleobase analogues [95]. Whereas, *ara*-2′-F-NMC modification showed thermal destabilization of – 2.8°C and – 6.9°C per modifications versus RNA and DNA [95].

In 2018, Manoharan and co-workers synthesized various NMC analogues containing 2′-F substituent with all the nucleobases (A, G, C, and U) **[**Figure 10**]** [96]. 2’-F-NMC had greater 3’-exonuclease and 5’-exonuclease stabilities when placed at the penultimate position in either 3ʹor 5’ ends of the oligonucleotides as compared to 2’-F RNA [96]. Recently, Manoharan and co-workers have evaluated gene silencing and off-target studies using 2’-F-NMC modified siRNA [97]. In addition, RNAi-mediated gene silencing was investigated using the 2’-F-NMC uridine containing 5’-*E*-VP modification, which was incorporated at the 5’-end of the guide strand **[**Figure 10**]** [97]. *In vitro* studies were carried out using 2’-F-NMC modified siRNAs targeting to the *Ttr* gene in primary mouse hepatocytes. The 2’-F-NMC at position 1 in the guide strand reduced the RNAi activity compared to the parent siRNA duplex.

Interestingly, it was found that the 2’-F-NMC with 5’-*E*-VP modification (5’-(*E*)-VP 2’-F-NMC) at position 1 of the guide strand of the siRNA is considerably less potent compared to 2’-F-NMC at position 1 with 5’-P combinations. The 2’-F-NMC modification was well tolerated at the 10, 11, and 12 positions of the guide strand. In addition, incorporation of two adjacent 2’-F-NMC modifications at the 22 and 23 positions of the guide strand of the siRNA with either PO or PS linkages was less potent than the parent siRNA. Incorporation of the 2’-F-NMC modification at positions 1, 4, and 19 of the passenger strands has shown similar potency compared to the parent siRNA.

*In vivo* studies using mice models showed that at position 1 of guide strand the 5’-(*E*)-VP 2’-F-NMC modification exhibited only 30% *Ttr* mRNA silencing. The 2’-F-NMC modification had similar gene silencing at positions 3, 10, or 11 as of parent. On the other hand, the 2’-F-NMC at position 7 exhibited an 80% reduction in TTR level on day 7. Off-target results illustrated that the NMC modification does not have a positive or negative impact on seed-mediated off-target activity [97]. Further *in vitro* and *in vivo* evaluation of these modifications is required to understand the full potential of 2’-F-NMC modification in siRNA.

## 4. Concluding remarks

siRNA therapeutics have emerged as one of the most powerful therapeutic tools in the last decade. After RNAi discovery, several siRNA-based drug molecules entered clinical trials. Four siRNA drugs including patisiran, givosiran, inclisiran, and lumasiran, have been approved in the last 3 years. In addition, several potent siRNA drug candidates for the treatment of various diseases are at advanced stages of clinical trials. Most of the approved siRNA drugs are completely or partially modified with 2’-OMe, F, and PS modifications that have improved nuclease stability and physicochemical properties. In recent years, emerging dual ribose modifications including 2ʹ4’, 2ʹ5’, 5’-*E/Z*-VP, and NMC modifications have been evaluated for RNAi activity.

Chemical modification at the C4’/C5’-ribose plays a crucial role in tuning the sugar conformation and stereoelectronic properties. The C5’-position modified with 5’-(*E*)-vinylphosphonate at the first position in the guide strand acts as a metabolically stable phosphate mimic which improves exonuclease stability and *in vivo* potency of siRNA. In general, most of these modifications exhibited enhanced nuclease stability, prolonged gene silencing efficiency, reduced off-target effects, and higher tissue accumulation and retention compared to 2’-modifications. Certainly, the close proximity of the 4’ and 5’ modifications to vicinal phosphodiester helps to improve the nuclease stability of siRNAs which results in prolonged silencing activity. From these results, it could predict that the synergistic effects of the minimal number of dual chemical modifications in sugar will be sufficient to achieve the desired siRNA therapeutic activity. However, most of the emerging dual ribose modifications studied so far need extensive comparative analysis in order to find the best compatibility with RNAi machinery. Therefore, an advanced chemical modification platform using the combination of these dual modifications is needed to improve the success rate of siRNAs in clinical trials.
